# General Education Teachers’ Perceptions of Autism, Inclusive Practices, and Relationship Building Strategies

**DOI:** 10.1007/s10803-021-05266-4

**Published:** 2021-09-21

**Authors:** Yasamin Bolourian, Ainsley Losh, Narmene Hamsho, Abbey Eisenhower, Jan Blacher

**Affiliations:** 1 Graduate School of Education, University of California, Riverside, 1207 Sproul Hall, Riverside, CA 95251 USA; 2 College of Liberal Arts, University of Massachusetts, Boston, Boston, MA 02125 USA

**Keywords:** General education, Inclusion, Autism spectrum disorder, Teacher perceptions, Pedagogical practices, Student–teacher relationships

## Abstract

To identify target areas for professional development, this mixed-methods study examined general education teachers’ perceptions of autism and pedagogical practices in early elementary classrooms in the United States. In focus groups, teachers (*N* = 18) identified terms they associated with autism and strategies they used for inclusion and relationship building. Participants systematically free-listed and ranked their responses to three prompts. Using ranked responses, saliency scores were calculated to assess the perceived importance and frequency of responses. Teachers’ most salient perceptions of autism (e.g., social difficulties, focused/fixed interests) revealed an awareness of core symptoms. Salient inclusion practices included assigning special classroom responsibilities and showcasing student talents; salient relationship-building strategies included embracing students’ special interests and engaging in one-on-one time. Implications for teacher trainings are discussed.

## Introduction

Research pointing to the academic and social benefits of inclusive educational placement has guided the evolution of education policy over the last forty-five years. As a result, many students with disabilities, including autism spectrum disorder (ASD), are being educated in inclusive environments, primarily in the general education setting. In 2015, about 91% of autistic students^1^ in the United States were in general education schools, and about 40% of these students were placed in general education classrooms for at least 80% of the day, alongside their neurotypical peers (U.S. Department of Education, 2019).

Once students with disabilities are placed in general education, their teachers play a critical role in enabling and facilitating inclusion practices that promote equal access to the curriculum, as well as to social opportunities in the classroom (Individuals with Disabilities Education Act, 1990, 2004). However, even though inclusion has been shown to support high levels of achievement for many students, often including autistic students (e.g., Ferraioli & Harris, 2011; National Research Council, 2001), the adoption of inclusive practices in integrated classrooms has lagged behind, highlighting the need for improved professional development around inclusive education practices for teachers (van Mieghem et al., 2020).

Research suggests that general education teachers may not be actively employing inclusion strategies in their classrooms. For example, in a large mixed-methods study, Segall and Campbell (2012) found that, while general and special education teachers similarly understood the importance of inclusion, general education teachers were less likely to report using best practices of inclusive education for students with disabilities. This study, as well as extant research, suggests that the effectiveness of inclusion is largely influenced by teachers’ opinions of disabilities and their receptivity towards inclusion (Avramidis & Norwich, 2002; Robertson et al., 2003; Segall & Campbell, 2012; Syriopoulou-Deli et al., 2012). That is, unfavorable perceptions of students with disabilities and/or inclusive practices may negatively impact teachers’ willingness to accommodate students with disabilities and their confidence to effectively integrate these students into classroom activities.

Teachers’ perceptions of ASD and inclusion appear to be shaped by both (1) their past or current experiences of autistic students, including the pervasiveness of child behavior problems and modification needs in the classroom, and (2) teacher variables, including training and perceived teaching efficacy (Avramidis & Norwich, 2002; Coman et al., 2013; de Boer et al., 2011; Forlin & Chambers, 2011; Hastings & Oakford, 2003; Van Reusen et al., 2001; Vaz et al., 2015). For example, teachers generally express more positive attitudes towards the inclusion of children with physical impairments than to children with emotional and behavioral difficulties (Avramidis & Norwich, 2002). Moreover, Van Reusen et al. (2001) found that among 125 general education teachers, respondents with more negative attitudes towards inclusion were those who had little knowledge or training in special education. Given that teachers’ perceptions can dramatically affect the successful integration of students with disabilities, it is important to understand these viewpoints to determine how to best support teachers in creating a beneficial learning environment for their students. The present study aimed to gather insights into teachers’ perceptions of ASD and their practices that affect inclusive education and student–teacher relationships in the early school grades.

### Autistic Students in Inclusive Classrooms

Autistic students in general education classrooms often demonstrate relative strengths that allow them to benefit from general education instruction. For example, in comparison to their counterparts in special education classrooms, autistic students in general education typically score higher on measures of intellectual functioning and communication skills (White et al., 2007). However, the prevalent characteristic features of ASD (i.e., varying degrees of social communication difficulties and repetitive patterns of behaviors or interests; American Psychiatric Association, 2013) can also pose challenges for teachers in inclusive classrooms.

Moreover, autistic students have increased susceptibility to emotional and behavioral difficulties, such as anxiety, emotion dysregulation, inattention, and disruptive behaviors (e.g., Blacher & Baker, 2019; Eisenhower et al., 2007; Leyfer et al., 2006; Samson et al., 2013). In particular, child behavior problems have been consistently shown to contribute to higher levels of teacher stress, lower levels of teaching efficacy, and poorer student–teacher relationship quality (Eisenhower., 2015; Herman et al., 2018, 2020). In a survey study of 655 general education teachers in K-12 grades, participants indicated that the greatest challenges of teaching autistic students were behavioral difficulties, as well as inappropriate social behaviors (Teffs & Whitbread, 2009). Thus, the inclusion of autistic students may feel daunting for general education teachers who have to adapt their usual practices to meet these social, emotional, and behavioral needs, often without sufficient support or training.

Past research shows that autistic students experience poor relationships with their teachers on average, marked by low closeness and high conflict (Blacher et al., 2014). This is important as the quality of student–teacher relationships (STRs) can serve as both a protective and predictive factor of student outcomes (e.g., Caplan et al., 2016; McGrath & Van Bergen, 2015), with poor quality STRs linked to poor school adjustment, increased child behavior problems, and increased social difficulties (Blacher et al., 2014; Doumen et al., 2008; Sette et al., 2013). Robertson et al. (2003) examined the relationships of 187 teachers and their students in second and third grade general education classrooms, including 12 autistic students. They found that when teachers had positive perceptions of their STRs, they reported lower levels of child behavior problems, and students had higher peer-rated levels of social inclusion. Therefore, when STRs are strong, teachers may tap into this relational strength to facilitate the social acceptance of autistic students in general education classrooms.

Among general education teachers responsible for autistic students, low levels of teaching efficacy are particularly pronounced, in part due to a lack of education and training in special education and ASD (Blacher et al., 2015; Blanton et al., 2011; Bocala et al., 2010; Kisbu-Sakarya, & Doenyas, 2021). Indeed, many general education teachers report feeling ill-equipped to effectively teach and positively interact with autistic students (All Party Parliamentary Group on Autism [APPGA], 2017; Roberts & Simpson, 2016; Scheuerman et al., 2003). A large body of teacher training research indicates that teacher knowledge of ASD is crucial for promoting positive school experiences. A study in the U.K. reported that fewer than half of 176 youth surveyed reported being happy at school; importantly, 60% expressed that the main factor that would make school better for them is having a teacher who understands their autism (APPGA, 2017). Notably, professional development training in ASD shows promise for increasing teachers’ knowledge and enhancing their perceptions, attitudes, and self-efficacy (Allday et al., 2013; Coman et al., 2013; Klassen et al., 2011; Leblanc et al., 2009; Loiacono & Valenti, 2010; Park & Chitiyo, 2010; Parsons et al., 2016; Syriopoulou-Deli et al., 2012; von Suchodoletz et al., 2018).

### Purpose of Present Study

In order to best support teachers of autistic children in general education settings (e.g., tailoring teacher training programs), we must learn more about teachers’ perceptions of ASD and their existing practices and strategies around including and connecting with autistic students. The present study is part of a larger project, funded by the Institute of Education Sciences, aimed at developing and implementing a professional development program on ASD for general education teachers in early elementary school (Kindergarten through 3rd Grade). Utilizing an iterative approach to program development, an initial step was to conduct focus groups to investigate the perspectives of general education teachers on the following: (a) their views of ASD, (b) strategies they use to effectively include autistic students, and (c) how they foster positive relationships with these students in the classroom. Based upon the findings from previous research (Roberts & Simpson, 2016), it was hypothesized that general education teachers would demonstrate somewhat negative opinions regarding autistic students and would report a range of general and specific responses on strategies for effective inclusion and enhanced relationships. Findings from these focus groups, combined with findings from existing research, were subsequently utilized to build the evidence base for the larger professional development program.

## Methods

### Participants

As part of the advancement of an autism-focused professional development program, general education teachers in Southern California and Massachusetts (USA) were invited to participate in a half-day (4-h) focus group to understand educator perceptions about topics central to autism. Eligible teachers had previously taught at least one autistic student in a kindergarten through third grade general education or inclusion classroom. Once eligibility was determined and study information was shared by phone, teachers reviewed consent forms via email; signed consent was obtained at the start of the focus group after the facilitators addressed any questions. Focus groups took place outside of the school day at a local school facility and a university center in California and Massachusetts, respectively. Each group consisted of two co-facilitators, a note-taker, and eight to ten participants. Teachers received honoraria for their participation.

At the onset of the focus group, participating teachers completed a demographic survey. Across study sites, 18 teachers participated (17 female, one male). Race was assessed with an open-ended item later aggregated into categories. Thirteen teachers identified as White, three as Asian, one as Black/African American, and one as multi-racial. Thirteen teachers had at least a master’s degree. Teachers had a mean of 14.9 years of teaching experience (SD = 12.2; range = 1–41). At the time of the focus group, teachers’ placements were in kindergarten (3), first (9), second (4), and third grade (2). All teachers taught at public schools, including six at public charter schools. All teachers were primary teachers or co-teachers in general education or inclusion classrooms; on average, teachers had 24 students in their classes and three students with Individualized Education Programs (IEPs). Five teachers had previously received some form of professional training in autism.

### Focus Group Design

A university institutional review board approved all study procedures. Three prompts were presented to the focus group to elicit teachers’ perspectives on the topics of interest: (1) perceptions of autism: “Think about the student or students with autism who have been in your classroom. What words or phrases come to mind when you think about this student or students with autism?”; (2) inclusion strategies: “In what ways have you effectively included children with ASD in your general education classroom settings?”; and (3) relationship-building: “Can you identify and describe any strategies or techniques that are particularly effective in developing relationships with your students with autism?”^2^ The terms “ASD” and “autism” were explained to teachers in the introduction and were used interchangeably during focus groups.

This paper presents a focus group design derived and adapted from Cohen and Miguel (2018) and Grinker et al. (2015). For each prompt, participants engaged in a series of three guided steps, including free-listing, ranking, and explanation of rankings. First (free-listing step), teachers were asked to write down words or word phrases on individual notecards with no identifying information. Teachers were given five notecards but were encouraged to use as many or few notecards as desired. All cards were then collected, shuffled to prevent identification, and collectively displayed on a table to all participants. Next (ranking step), participants were asked to review the displayed cards and individually identify the top five cards of the entire collection in order of importance (e.g., rank the top five words/phrases that best describe ASD in your opinion, “1” being the most important) on a piece of paper. Participants could select and rank the cards from any teacher, not only their own. Lastly (explanation of rankings step), teachers were asked to explain their rankings, and the rationale for those rankings, aloud. This process was repeated for each of the three prompts. Focus groups were recorded and transcribed verbatim.

### Analyses

The analytic plan and coding process for this study, demonstrated in Table 1, was adapted from Cohen and Miguel (2018) and Grinker et al. (2015). As a first step in the qualitative analysis, two coders compiled and reviewed the free-list responses. Synonymous or similar responses within each prompt were identified and combined into categories. For example, in response to the first prompt, “difficult peer interactions” and “social difficulties” were grouped into one category (“Difficulty with Social Interactions”). Each response that contained more than one distinct content area was categorized as a separate unit. Thus, categories maintained the accuracy of individual responses, while representing the range of distinct responses. Each of the two coders initially coded all responses independently for synonymous words or phrases; the two coders then met to discuss discrepancies until agreement was reached. For responses that needed clarification or context, coders referred to the explanation of rankings step in the focus group transcripts, in which participating teachers explained their ranked responses aloud.

**Table 1:** Coding process followed by two coders

Independent review of free-list responses	Independent identification of categories across similar/synonymous responses^a^	Coders collaborate to reach consensus on categories	Calculate saliency scores for each response category^b^
280 total free-list responses		86 total response categories	

^a^For responses that needed clarification or context, coders referred to the explanation of ranking step in the focus group transcripts, in which participating teachers explained their ranked responses aloud

^b^Tables 2, 3 and 4 show response categories and respective saliency scores

There were 29 response categories for the first prompt, 29 for the second prompt, and 28 for the third prompt. Within each prompt, saliency scores were calculated for all categories in order to distinguish the relative degree of salience, or prominence, among categories (Cohen & Miguel, 2018; Grinker et al., 2015). To calculate saliency scores, a two-step process was employed. First, each ranked response was reverse scored, such that a ranking of 5 (least important) was replaced with a numeric value of 1; a ranking of 4 was replaced with a 2, and so on. Reverse scores for ranked responses were then summed and divided by the total number of participants (N = 18) to create an average score per category. Categories that were ranked by more participants earned higher scores. Thus, higher saliency scores indicated responses that were more often ranked as important and, when ranked, were more often ranked highly, reflecting greater consensus across participants (Cohen & Miguel, 2018; Grinker et al., 2015). The process of calculating saliency scores for response categories is demonstrated in Table 2.

**Table 2:** An example of the saliency score calculation (conducted for each response category per prompt)

Response category	Individual written responses	Ranking–reverse code	Calculation^a^	Saliency score
“Social disconnect”	Disconnected	1–5	34/18	1.89
	Disconnected	1–5		
	Disconnected	3–3		
	Disconnected (or connected alternatively)	2–4		
	Lacks social skills/**disconnected**	1–5		
	Socially disconnected	1–5		
	Socially disconnected	2–4		
	Socially disconnected	3–3		

Bold text denotes coded text where more than one unique response was present

^a^Saliency calculation = Sum of reverse codes/sample size

## Results

The results, presented below, highlight the five most salient categories for each focus group prompt. For each category, saliency scores are provided in parentheses and accompanied by illustrative quotes.

### Teacher Perceptions of Autistic Students

The first prompt yielded 29 distinct response categories on teachers’ impressions of autistic students, with saliency scores ranging from 1.89 to 0.05. Response categories for Prompt 1 are provided, in order of most to least salient, in Table 3. Overall, the highest saliency terms did not express negative perceptions of ASD; rather, they reflected teachers’ understanding of common social and behavioral traits that are often characteristic of autistic students. The five most prominent categories were: *Social Disconnect*, *Sensory Sensitivities*, *Difficulty with Social Interactions*, *Emotion Dysregulation*, and *Focused or Fixed Interests*. The below quotes illustrate how teachers described each of these most salient perceptions of autism:

**Table 3:** Response categories and saliency scores for Prompt 1

*PROMPT 1* Think about the student or students with autism who have been in your classroom. What words or phrases come to mind when you think about this student or students with autism?	
*Response Categories* Teachers’ verbatim responses were aggregated into these categories	Saliency
Social disconnect	1.89
Sensory sensitivities	1.72
Difficulty with social interactions	1.56
Emotion dysregulation	1.39
Focused or fixated interests	1.33
Routine-based/structure-driven	1.22
Impulse control	0.89
Loveable	0.67
Lack of eye contact	0.56
Enjoyment	0.44
Difficult to redirect	0.39
Lack of social skills	0.33
Charming	0.33
Challenging behavior	0.33
Aggression	0.28
Easily frustrated	0.22
Observant	0.22
Desire to belong	0.22
Volatile	0.22
Egocentric	0.22
Fun-loving	0.17
Special	0.17
Intelligent	0.11
Strange behavior	0.11
Active	0.11
Unresponsive	0.11
Physical	0.11
Sweet	0.06
Strong willed	0.05

#### Social Disconnect (1.89)

“The ‘social disconnection.’ I struggle with that as I see them [autistic students] wanting to fit in, 'Will you be my friend? Do you want to play?’ And the other kids are like, 'No, no I don't.’ And you know, it's heartbreaking. So, I see them struggling socially, to socially connect, and I struggle with how to help them.”

#### Sensory Sensitivities (1.72)

“The children [on the autism spectrum] that I've had the pleasure of teaching have either been wanting to touch everything, which can cause a lot of problems with other students and leads to a lot of misunderstanding from adults. Or, they don't want to touch anything at all. They want to be away from everyone, which also causes a lot of misunderstandings.”

#### Difficulty with Social Interactions (1.56)

“Across the board, for all of the students who I've had in my classroom who are on the autism spectrum, difficulty socializing has been a very big prominent thing with them, and something that I've spent a lot of time trying to focus on because I've really noticed peer interactions are tough.”

#### Emotion Dysregulation (1.39)

“This [ranked response] was all about impulse control, what happens when structure is broken, with regulating emotions and outbursts and behaviors. The difficulty to deescalate once they've already escalated.”

#### Focused or Fixated Interests (1.33)

“On Thursday, we had someone in the classroom get sick, and then that student [on the autism spectrum] for the rest of the day kept saying, ‘Why did she get sick? Why did she throw up? Where did she go? What's happening?’ It's like, we don't need to talk about it! Just very fixated on things and hard to move on.”

### Classroom Practices for Inclusion

The second prompt regarding ways teachers have effectively included autistic students in the classroom yielded 29 distinct response categories, with saliency scores ranging from 1.56 to 0.06. Response categories for Prompt 2 are provided, in order of most to least salient, in Table 4. The five most prominent categories were: *Job Responsibilities*, *Showcasing Special Talents and Strengths*, *Visual Aids*, *Partner/Group Activities*, and *Classroom Relationships*.

**Table 4:** Response categories and saliency scores for Prompt 2

*PROMPT 2* In what ways have you effectively included children with ASD in your general education classroom or schooling settings?	
*Response Categories* Teachers’ verbatim responses were aggregated into these categories	Saliency
Job responsibilities	1.56
Showcasing special talents and strengths	1.50
Visual aids	1.06
Partner/group activities	0.94
Classroom relationships	0.83
Whole-class/group strategies	0.78
Modeling	0.78
Consistent supports and language use	0.78
Compliments	0.72
Routines	0.72
Positive feedback	0.72
Clear expectations and structure	0.61
Family/home relationships	0.61
Utilizing student interests	0.56
Sensory support	0.44
Chunking	0.33
Structured choice	0.28
Breaks	0.28
Educate others	0.28
Routinely monitor	0.28
Concrete goals linked to interest-based incentives	0.28
Seating arrangement	0.22
Time spent with teacher/peers	0.22
Patience	0.22
Parent–Teacher relationship	0.22
Movement	0.11
Proximity to teacher	0.11
Provide task support	0.06
Role play	0.06

#### Job Responsibilities (1.56)

The most salient category (*Job Responsibilities*) suggests that teachers highly endorse building a sense of student ownership and active participation in the classroom community. Focus group transcripts revealed that specific class jobs included taking down chairs in the morning, setting up laptops, passing out papers, and sharpening pencils. Teachers used these types of classroom roles to support student engagement, motivation, and attention, particularly during transition periods. Moreover, teachers expressed that when all students were assigned a class job, autistic students were more included.

“Because my little angel right now is super routine-driven, giving him a way to participate, to use that skill in a positive way. So, they feel like they have ownership of the classroom and they're a part of it.”

#### Showcasing Special Talents and Strengths (1.50)

“[Autistic student] was constantly eating her own hair, cutting her own hair, shoving it in her mouth, and it's really gross. Other kids didn't like that. But in my class, she actually remembered everybody's birthday. She had a remarkable memory...So I really emphasized [this strength]. Then, other students did not dislike her as much, even though she was shoving hair in her mouth every day.”

#### Visual Aids (1.06)

“My current student [on the autism spectrum], she has a visual schedule that's Velcro, and so I always organize that to reflect exactly what's going to happen in the day, and if there's a special event, we have a special card for special event, and I always kind of preview that with her when we walk into the classroom in the morning and that's very helpful. That's the strategy I wish I had used in the past with previous students [on the autism spectrum] because it's been really successful with her.”

#### Partner/Group Activities (0.94)

“I find that there are some kids in my class that are really great at working [together], particularly with the student [on the autism spectrum] I have this year, and some that are not. There's one girl in my class now, she's his partner when we do turn and talks on the rug, and she's picked up on me using consistent language, and so now she'll be like, ‘That's unexpected,’ or ‘That's off topic.’ But it's really great because she's meeting him where he is and not doing it in a condescending way... Often I'm catching them giggling when they should be doing work, which I want them to do their work, but that wasn't something I was seeing at the beginning of the year.”

#### Classroom Relationships (0.83)

“‘Building relationships’ is just having special time with teachers and with maybe a couple peers who you could select. I have done in the past like lunch groups, like grabbing that student and maybe one or two other kids for lunch, and having them eat in the classroom with me, and just giving them time to build those relationships in a little bit of an unstructured way. Like you're not working on this one math problem together. You can just kind of talk with me, helping you kind of figure out what's appropriate to ask your friend and what to talk to your friend about.”

### Strategies for Improving STRs

The third prompt surrounding strategies that effectively promote positive STRs with students yielded 28 unique response categories, with saliency scores ranging from 3.06 to 0.06. Response categories for Prompt 3 are provided, in order of most to least salient, in Table 5. The five most salient categories were: *Taking an Interest in Student Interests*, *Having One-On-One Time*, *Providing Safety, Being Patient,* and *Positive Feedback and Compliments*.

**Table 5:** Response categories and saliency scores for Prompt 3

*PROMPT 3* Identify and describe any strategies or techniques that are particularly effective in developing relationships with your students with autism	
*Response Categories* Teachers’ verbatim responses were aggregated into these categories	Saliency
Taking an interest in student interests	3.06
Having one-on-one time	1.33
Providing safety	1.33
Being patient	1.28
Positive feedback and compliments	1.11
Getting to know and interacting with the family	0.94
Meaningful interactions	0.72
Consistency	0.72
Being supportive	0.44
Proximity	0.44
Listening	0.39
Greeting	0.39
Accepting of differences	0.39
Personal attention	0.39
Consistent language	0.28
Special assistance	0.28
Acknowledging emotions	0.28
Playing/interacting with students on the playground	0.28
Visual aids	0.22
Understanding of student needs	0.22
Consistent interaction	0.17
Initiate play/conversation	0.17
Class meetings to talk about autism	0.17
Build relationships	0.17
Taking on student perspectives	0.11
Routines	0.11
Preferential seating	0.06
Eye contact	0.06

#### Taking an Interest in Student Interests (3.06)

*Taking an Interest in Student Interests* had a saliency score substantially higher than that of other categories, both within and across prompts. Within this category, focus group transcripts revealed that teachers endorsed making time to find their students’ interests, showing an interest in their likes, and celebrating their talents, as well as sharing about themselves.

“We had a student a few years ago who was obsessed with Thomas the Train. We gave him color sheets of Thomas as a reward. One time we gave him a big chart paper, and we said draw Thomas - just little things like that... Letting him build with Thomas Building Legos. We built a massive thing of Thomas, and he had it on window sill. He must have had forty [Thomas objects]. It was beautiful. He just loved it. He was amazing, and he showcased it.”

#### Having One-On-One Time (1.33)

“Finding time for one-on-one time, whether it be just like during snack, sitting with that particular student and talking more with them than with the other kids for that day. Just making sure you’re finding time for the positive time, especially if they have been struggling behaviorally in your class and it's starting to feel more negative. Always finding time in the day to have that like funny or joyful one-on-one time with them.”

#### Providing Safety (1.33)

Teachers endorsed the importance of providing a sense of safety and trust for the autistic child, for example by being consistent and predictable, as well as creating a literal safe, comfortable space or quiet corner within the classroom:

“Provide a safe place and then it's understood as time goes on throughout the year that it doesn't have to be guided for them. They know they can go over [to the safe place] and they can sit there when it's time for everybody to do writing or something that they get distracted or struggle with.”

#### Being Patient (1.28)

“Having patience and also just like understanding that sometimes it takes time to build relationships with these kids [on the autism spectrum]. They won’t come quickly.”

#### Positive Feedback and Compliments (0.94)

“My second [ranked response] that I chose was ‘positive feedback’ because these kids [on the autism spectrum] don’t fall into the same social circles as other kids do, so they may not be motivated by someone asking them to come over after school—that might be scary to them. But they still, like any other kid, they want positive feedback, whether that’s from us or from their peers. One idea that I really, really loved was this idea of a compliment circle. Just this idea that you’ve got all of these kids spreading that positivity with each other. I really love that idea.”

## Discussion

The purpose of this study was to examine general education teachers’ perceptions of ASD and their day-to-day educational practices for both inclusion and relationship-building with their own autistic students. The results of the mixed method analysis offer guidance for future professional development programs aimed at promoting best practices in the general education classroom, especially for autistic students. Notably, nearly three-fourths of participants indicated that they had not participated in autism-specific training in the past, highlighting a critical need.

### How Did General Education Teachers Describe Autistic Students?

Teachers generated a large number of unique responses to the first prompt, likely reflecting the fact that each autistic child presents a unique combination of symptoms and levels of severity. Perhaps counter to expectations given the literature on teachers’ opinions of the social and behavioral challenges of ASD (Teffs & Whitbread, 2009), in the current study, teachers’ descriptions of their autistic students were not predominantly negative or positive. Rather, their perceptions tended to be centered on observable behaviors that are characteristic of ASD (e.g., *Difficulty with Social Interactions*, *Focused or Fixated Interests*), suggesting that teachers possessed a good understanding of the hallmark features of ASD. The commonly endorsed individual responses (e.g., “difficulty with peers/socializing”, “fixated on certain ideas/rigid”) reflect themes that closely align with the diagnostic definition of ASD (APA, 2013), suggesting an accurate awareness of autism.

Despite this awareness of autism-related behaviors, a previous systematic review by Roberts and Simpson (2016) suggested that general education teachers may feel that they have limited knowledge of autism and relevant teaching strategies to support students on the spectrum. For this reason, affirming and expanding upon teachers’ understanding of ASD may promote their confidence and self-efficacy in working with autistic students in inclusive settings. Moreover, two of the most prominent categories within this prompt were *Social Disconnect* and *Difficulties with Social Interactions.* Certain evidence-based strategies can be utilized to support the understanding of social situations for autistic students in inclusive classrooms. Social narratives, for example, are tools that include both text and visual aids to describe social situations and to help autistic students better understand social cues (Steinbrenner et al., 2020). Social Stories ™ is one manualized social narrative intervention that has been tested for use as a “classroom friendly” intervention for teachers of autistic students in inclusive classrooms (Chan et al., 2008; Chan et al., 2011). Peer-mediated strategies (i.e., involving a peer to support and help engage autistic students in social situations) may also be an efficacious approach to promoting prosocial behaviors (Steinbrenner et al., 2020).

Teachers’ focus on observable student behaviors may promote more effective communication with colleagues (e.g., behavior specialists, paraprofessionals, special education teachers, school psychologists), as well as parents, when supporting students’ social and behavioral functioning. Further, unlike internal child attributes, these observable behaviors may be more malleable in response to teacher intervention, including positive behavior supports, instructional support, or relational strategies in the classroom. For example, antecedent-based strategies, such as using student preferences, changing schedules, and modifying the environment, have been shown to decrease challenging behavior and increase engagement (Steinbrenner et al., 2020). However, the effectiveness and selection of appropriate strategies is dependent upon defining observable behaviors as intervention targets. Thus, identifying and highlighting these characteristics of autistic students may lead general education teachers to be more effective, empowered, and self-efficacious in working with students (Wiley et al., 2012).

### What Inclusive Strategies were Endorsed by General Education Teachers?

General education teachers reported several strategies that they perceived to increase success for autistic students in inclusive classrooms, considering both academic and socioemotional outcomes. Assigning autistic students special jobs or responsibilities was the most salient strategy mentioned for promoting inclusion. By allowing students to share classroom responsibilities, the students have the opportunity to become an integral part of the classroom routines and management (Garrett, 2008). Indeed, in a study of 136 young children without autism (3–6 years), Bryan et al. (2014) found that being called a “helper” had implications for children feeling valued and developing positive identities. Autistic students may benefit from specific classroom role assignments as these roles may motivate them to engage in further prosocial behaviors. In addition, participating teachers highly endorsed the use of visual tools, such as reminders, schedules, and timers. These strategies are backed up by research indicating that visual supports can be effective for students who have difficulties processing auditory instruction and information, including those on the autism spectrum, and can be utilized to target many academic skills and behaviors, including task engagement and transitions across activities (Steinbrenner et al., 2020).

Teachers conveyed an understanding for the importance of predictable patterns and consistency, for example establishing routines, having clear expectations, and using consistent language (e.g., *Routines, Consistent Supports*, and *Language Use*). A reliance on classroom routines provides structure for autistic students, and in turn, students may be more likely to be engaged in learning and less likely to demonstrate behavior problems. Overall, these strategies may promote smooth classroom operations. Strategies also reflected teachers’ considerations of the physical classroom environment, such as space for movement, purposeful seating arrangements, and proximity to the teacher (e.g., *Seating Arrangement*; *Proximity to Teacher*), though these were not very salient. By strategically structuring the classroom layout, learning through various instructional activities (e.g., whole group, small group) may be more effectively facilitated, and disruptions to students may be minimized. Although proactive, antecedent-based strategies are evidence-based for promoting skill learning and prosocial behaviors for autistic students (Steinbrenner et al., 2020), some of these strategies had low saliency scores, suggesting a possible area to emphasize in future teacher trainings.

Lastly, the prompt elicited responses about family relationships and collaborations (Family/Home Relationships, Parent-Teacher Relationships), though these strategies had relatively low saliency scores of 0.61 and 0.22, respectively, suggesting that teachers may not necessarily view family-school partnerships as addressing inclusion. The lack of endorsement for family collaboration supports previous research demonstrating teachers’ dissatisfaction with the level of parental involvement (Lindsay et al., 2013). Previous literature has underscored the importance of home-school connections and parent-teacher relationships for student outcomes and inclusion (e.g., academic engagement, social skills; Eisenhower., 2015; Roberts & Simpson, 2016; Serpell & Mashburn, 2012). Thus family-school partnerships may be another area to emphasize in teacher interventions promoting inclusion.

Strategies related to praise and positive appraisal (e.g., engaging the class in compliment circles, providing positive feedback, and capitalizing on special talents/strengths) were also reported as means of promoting inclusion, with varying levels of prominence. *Showcasing Special Talents and Strengths* was among those highly endorsed. In one study (Saggers et al., 2011), nine autistic students enrolled in a mainstream high school (ages 13–16) were interviewed to examine their perspectives and experiences of inclusive education. Based on student narratives, several impactful areas emerged (e.g., teacher characteristics, curriculum-related issues, friendships). Notably, students viewed teacher characteristics as being most crucial to school life and expressed that teachers’ understanding of individual strengths, as well as active listening, significantly contributed to successful inclusion. Thus, both teachers and older autistic students recognized the importance of incorporating student talents and strengths as an inclusive strategy. In their systematic review of the perspectives of stakeholders on the inclusion of autistic students, Roberts and Simpson (2016) found mixed results surrounding teachers’ opinions of utilizing students’ unique interests to promote inclusion, with some teachers finding it helpful for autistic students to be viewed by peers as “experts” in areas of interest and some finding it a barrier to peer relationships (i.e., hyper-focus on preferred subject(s) viewed as odd). Taken together, it may be important to address some of these perceived barriers to highlighting autistic students’ unique talents, strengths, and interests in inclusive settings (e.g., teaching peers to be inclusive, understanding, and accepting of differences).

While showcasing students’ special talents and strengths was identified as one of the more salient strategies for inclusion by teachers in the present study, strengths and positive attributes of ASD (e.g., *Loveable, Charming*) were not highly ranked in response to the first prompt assessing teachers’ perception of autistic students. This pattern suggests that, in order to effectively highlight students’ strengths and talents, teachers may need help identifying the unique strengths and positive characteristics of their autistic students. Furthermore, endorsed inclusive strategies reflected a certain degree of individualization, for example *Utilizing Student Interests* and *Concrete Goals Linked to Interest-Based Incentives*. This suggests that teachers are aware of, and are harnessing, the power of student special interests, either as a reinforcement or as a tool for engaging them in the curriculum, and this is promising.

### How did General Education Teachers Promote Positive Relationships with Their Autistic Students?

Strategies endorsed by teachers to build relationships with their autistic students ranged from broad, general strategies, such as being consistent, listening, and/or being patient, to more specific, concrete strategies, such as greeting students by name at the door or having one-on-one time. Responses suggested that teachers aim to develop positive STRs through (1) increased openness, warmth, and closeness between the student and teacher (e.g., *Taking an Interest in Student Interests; Providing Safety; Being Supportive; Accepting of Differences*), (2) positive behavioral supports (e.g., *Positive Feedback and Compliments; Consistency*), and (3) homeschool connections/collaboration (e.g., *Getting to Know and Interacting with the Family*). Each of these elements has indeed been associated with positive STR quality in previous studies (Allen et al., 2011; Baker et al., 2008; Dearing et al., 2008; Myers & Pianta, 2008). Thus, the present results suggest that research findings are reflected in teachers’ applied practices.

Importantly, despite the awareness of these strategies demonstrated by participating teachers, autistic students are generally more at-risk for poorer-quality STRs than both neurotypical students and students with other disabilities (Blacher et al., 2014; Longobardi et al., 2012). The majority of the strategies that were endorsed by teachers are not specific to autistic students in their effectiveness, and although this is advantageous in that the strategies may be universally beneficial to all students, some of the endorsed strategies may require additional adaptations to be effective and accessible with autistic students. For example, while *Taking an Interest in Student Interests* was the most salient relationship-building strategy, autistic students may have difficulty with social reciprocity, including sharing or reporting on their experiences. In addition, students’ ASD-related traits, such as difficulty with reciprocity, stereotyped speech, restricted interests, or often singular focus on a topic of interest, may make these conversations more challenging for teachers. Thus, professional development may need to focus on preparing teachers with more specific expectations and adaptations within these broader, well-known STR building strategies to best support their autistic students, specifically.

Moreover, teachers infrequently endorsed listening (i.e., “active listening”, “listen to needs”) as a strategy to develop STRs. Active listening involves making empathetic comments, asking appropriate questions, and summarizing for verification (Gordon, 2003). Research using student perspectives indicates that listening can be a powerful way for teachers to build high-quality relationships with students (Cefai & Cooper, 2010; Johnson, 2008), including those on the autism spectrum (Gray & Donnelly, 2013; Saggers et al., 2011). Utilizing a case study design, Gray and Donnelly (2013) engaged in general discussions with 12 autistic children (ages 4.6–7.8 years), using a range of prompts, about their likes and dislikes with regards to school. One of the older autistic children (aged 7 years) reported more positively about school because his teacher was helpful and listened to him. In another study of autistic high school students (Saggers et al., 2011), active listening was identified by students as a positive teacher characteristic as it allowed them to be understood. Therefore, teachers may find that engaging in student-centered discussions and reflective social interactions with autistic students can be effective in fostering STRs. Studies have also shown that active listening training can lead to improvements in teachers’ communication skills, as well as increased preparedness towards interaction with parents (McNaughton & Vostal, 2010; McNaughton et al., 2008).

## Future Directions and Limitations

Findings from this study provide useful teacher-reported, classroom-based information that can be utilized in the development and modification of teacher training programs. While resources for practitioners who work with autistic students, such as the Autism Focused Intervention Resources and Modules (AFIRM, 2018) by the NPDC, exist, teachers in this study generated a wealth of rich responses that should inform professional development programs going forward. For example, the practices reported in this study could be incorporated into future trainings as a reflection of strategies that, by nature of their endorsement by teachers, are likely to be viewed as acceptable and feasible in the classroom. Those practices that were both (1) reported by teachers to be highly salient and (2) supported by previous literature to be effective for autistic students may be the most beneficial to emphasize in teacher training programs. In addition, strategies that are considered evidence-based for supporting autistic children, but that were not highly endorsed by teachers, may indicate practices with barriers to implementation that should be further explored in future research and addressed in teacher training programs. On the other hand, strategies that were highly endorsed by teachers and that are aligned with existing evidence-based practices may be prime targets for future training programs. Based on teacher generated responses in this study, the following are considerations for future programming:

1. Teachers demonstrated a strong understanding of the observable characteristics of ASD. Ensuring that teachers can identify not only these observable characteristics (e.g., social disconnect, focused or fixated interests), but also students’ unique strengths and interests, may help promote more effective communication with colleagues and parents of autistic students.
2. The importance of recognizing student talents, strengths, and interests was commonly discussed by teachers as important for promoting inclusion and positive student–teacher relationships. At the same time, when asked to describe their autistic students, teachers were relatively less able to identify strengths, tending to name negative rather than positive attributes of autism. Thus, teachers may need greater support around specific ways to identify and effectively highlight the unique skills, strengths, and interests of their autistic students.
3. In their perceptions of autistic students, some teachers endorsed challenging classroom behaviors (e.g., impulse control, difficult to redirect, aggression). This aligns with previous research that suggests general education teachers have substantial concerns about autistic students’ challenging behavior (Roberts & Simpson, 2016). To provide teachers with support in addressing these behaviors, evidence-based behavior management strategies may be beneficial for general education teacher-focused training programs on autism. Some evidence-based behavior management strategies that were endorsed by teachers for promoting inclusion and positive relationships included positive reinforcement (e.g., praise, positive feedback, interest-based incentives) and antecedent-based strategies (e.g., routines, clear expectations, and structure).
4. Assigning special jobs or responsibilities was highly regarded as a tool for building an inclusive classroom environment. This may be a feasible (and possibly effective) strategy to foster inclusion, especially in the context of a classroom where students are often given special roles or duties.
5. Teachers may not often consider the physical classroom environment as part of an inclusive educational setting. Some physical factors (e.g., space for movement, purposeful seating, proximity to the teacher) can be addressed as proactive strategies to make inclusion easier.
6. Teachers recognized the role of home-school collaboration and communication for building positive relationships with autistic students but viewed this as less salient for promoting inclusion overall. Teacher training programs should emphasize the relevance of parent-teacher relationships for promoting the successful inclusion of autistic students more broadly (e.g., solicit parents’ help in identifying students’ unique talents, strengths, and interests; promoting students’ academic engagement).
7. Teachers identified “ordinary” daily classroom practices (e.g., consistency, greeting students by name at the door) that could enhance STRs with autistic students. Yet, teachers may need greater support on how to make time for and implement these on an everyday basis.
8. Training in active listening may promote greater understanding between teachers and students, as well as between teachers and parents.

As with any study, it is important to interpret these findings and considerations within the context of its limitations. The teachers who participated in this study were drawn from two regions of the United States. Thus, we recognize that their views may not be representative of all early elementary general education teachers across the United States or even internationally, particularly as inclusive practices are difficult to compare at an international level (D’Alessio & Watkins, 2009). In addition, teacher participants were volunteers who may have been more aware of and/or interested in autism than most. Nevertheless, nearly 75% of participants had not previously completed any training on ASD. Despite these potential limitations to the generalizability of results, the emphasis on and careful consideration of teacher voices is a notable strength. Involving teachers to generate concrete ideas is a promising means of identifying strategies that are likely to be perceived as feasible and acceptable in the classroom, thereby making this a particularly useful methodology for informing the development of teacher training.

## Footnotes

1 Identity-first language (i.e., “autistic person”) or “person on the autism spectrum” are used in place of person-first language (i.e., “person with autism spectrum disorder”) to reflect emerging perspectives and preferences from the autistic community (e.g., Botha et al., 2021; Bottema-Beutel et al., 2020; Kenny et al., 2016).

2 During focus groups, we used person-first language in line with common educational parlance at the time. The research team has since changed our study terminology to better reflect the preferences of most autistic adults.
